# Regulatory B and T lymphocytes in multiple sclerosis: friends or foes?

**DOI:** 10.1007/s13317-018-0109-x

**Published:** 2018-11-10

**Authors:** Georgios K. Vasileiadis, Efthymios Dardiotis, Athanasios Mavropoulos, Zisis Tsouris, Vana Tsimourtou, Dimitrios P. Bogdanos, Lazaros I. Sakkas, Georgios M. Hadjigeorgiou

**Affiliations:** 1 Department of Neurology and Laboratory of Neurogenetics, Faculty of Medicine, School of Health Sciences, University General Hospital of Larissa, University of Thessaly, Biopolis, 40500 Larissa, Greece; 2 Department of Rheumatology and Clinical Immunology, Faculty of Medicine, School of Health Sciences, University General Hospital of Larissa, University of Thessaly, Biopolis, 40500 Larissa, Greece; 3 Department of Neurology, Medical School, University of Cyprus, 1678 Nicosia, Cyprus

**Keywords:** Autoimmunity, Demyelination, Immunity, Regulation

## Abstract

Current clinical experience with immunomodulatory agents and monoclonal antibodies in principle has established the benefit of depleting lymphocytic populations in relapsing–remitting multiple sclerosis (RRMS). B and T cells may exert multiple pro-inflammatory actions, but also possess regulatory functions making their role in RRMS pathogenesis much more complex. There is no clear correlation of Tregs and Bregs with clinical features of the disease. Herein, we discuss the emerging data on regulatory T and B cell subset distributions in MS and their roles in the pathophysiology of MS and its murine model, experimental autoimmune encephalomyelitis (EAE). In addition, we summarize the immunomodulatory properties of certain MS therapeutic agents through their effect on such regulatory cell subsets and their relevance to clinical outcomes.

## Introduction

### Multiple sclerosis

#### Overview

Multiple sclerosis (MS) is an inflammatory disorder of the brain and spinal cord characterized by focal lymphocytic infiltration and microglial activation leading to neurodegeneration and progressive disability [1].

MS is the most common chronic neurological disease in young and middle-aged adults, affecting 2.5 million people worldwide. It is more prevalent in Northern Europe, Canada and Oceania and shows a female preponderance, with a female-to-male 2:1 ratio. MS is classified into three subtypes, namely relapsing–remitting MS (RRMS), primary progressive MS (PPMS) and secondary progressive MS (SPMS). RRMS, which accounts for 80% of the patients, presents at first with an acute episode affecting one or more sites, known as the clinically isolated syndrome (CIS). A second attack of demyelination occurring afterward is required to meet the diagnostic criteria for RRMS. Ultimately, around 65% of RRMS patients enter the SPMS phase, while in 20% the illness is progressive from onset, hence the characterization as PPMS [1].

The first therapeutic regimens that became available for MS were interferon-β and glatiramer acetate [2–4]. The FDA has currently licensed several immune disease-modifying therapies (DMTs), most of which have been validated in other autoimmune diseases as well. These include monoclonal antibodies, such as rituximab (anti-CD20) [5] and alemtuzumab (anti-CD52) [6, 7], and oral agents with immunomodulatory properties, such as fingolimod, dimethyl fumarate and teriflunomide [8–10]. Clinical trials testing anti-CD20-mediated depletion of peripheral B cells showed promising effects against the development of new central nervous system (CNS) lesions and relapses [11, 12]. Despite some efficacy of therapies, there are still unmet therapeutic needs in MS. Besides, current therapeutic agents are costly summing up to a total annual cost of approximately 15 billion euros for MS in Europe in 2010.

#### Pathogenesis

Environmental, genetic, epigenetic and immunological factors are implicated in the development of MS [13, 14]. Myelin-targeting autoreactive CD4(+) T cells that pass through a disrupted blood–brain barrier (BBB) and enter the CNS were initially considered the critical orchestrators in the pathogenesis of MS [15]. Activated by microglia, astrocytes or other immune cells through HLA class II presentation of myelin antigens, CD4(+) T cells express different cytokines depending on their subset. Th1 and Th17 cells express pro-inflammatory cytokines, such as IFN-γ and IL-17, respectively, whereas Th2 and regulatory T cells (Tregs) produce anti-inflammatory cytokines, such as IL-10 [16–18]. Thus, the skewing toward Th1 and Th17 responses is responsible for the immune-mediated damage of myelin and axons [19].

The activation of CD4(+) T cells within the CNS leads to the recruitment of other inflammatory cells, such as B cells, which cross the BBB, undergo activation, antigen-driven affinity maturation and clonal expansion [20]. In recent years, accumulated evidence emphasizes the role of B cells in the progression of MS [21, 22]. B cells are especially efficient in presenting antigens to CD4(+) T cells through HLA class II molecules [23]. Apart from antigen presentation, B cells are also able to produce autoantibodies after differentiation to plasma cells. Autoantibodies are able to cause demyelination through antibody-dependent cellular cytotoxicity (ADCC) and complement activation. Lastly, B cells are able to express pro-inflammatory cytokines, such as IL-6, IFN-γ and TNFα, known to be implicated in MS [24–26]. In particular, RRMS patients were found to have elevated peripheral expression of IFN-γ, interleukin (IL) 1-beta (IL1B), IL-7, IL-10, IL12A, IL-15, IL-23, IL-27, lymphotoxin-alpha (LTA) and lymphotoxin-beta (LTB) [27]. Τhe main sources of pro-inflammatory cytokines within PBMCs were T- and B cells, whereas monocytes were the most noticeable source of immunoregulatory cytokines [27]. Hence, the inflammatory reaction of T, B and other immune cells leads to demyelinated lesions throughout the CNS [28]. Interestingly, healthy individuals also possess autoreactive T cells at lower frequencies, a finding which signifies that their presence is not enough per se for disease induction [29]. Thus, healthy individuals are likely to maintain regulatory mechanisms that keep these autoreactive T cells under control. Emerging evidence suggests that both Tregs and Bregs play a major role in this ‘safeguard’ process.

#### Tregs

Tregs were originally identified by Sakaguchi et al. in 1995 as a CD4(+)CD25(+) T cell subset with suppressive activity [30]. They are essential to the maintenance of self-tolerance and their impairment has been linked with autoimmunity and includes numerical decreases, functional defects and conversion into inflammatory effector cells [31]. High expression of CD25 and low expression of CD127 are the main phenotypic markers characterizing bona fide human Tregs [32]. CD25 (IL-2 receptor) is central to Treg ontogeny, optimal regulatory function and proliferation mediated by the gamma chain cytokines IL-2, IL-4, IL-7 and IL-15 [33]. Subsequently, it was shown that cells with regulatory capacity can also express CD8 [34], cytotoxic T-Lymphocyte antigen-4 (CTLA-4) [35, 36], the TCR-inducible co-stimulatory receptor (ICOS) [37] and high levels of CD5 [38, 39], a surface marker that instructs extrathymic Treg cell development in response to self and tolerizing antigens also co-expressed by certain B regulatory cell subsets [40, 41] (as discussed below). The seminal discovery of forkhead box P3 (FoxP3), as a fundamental transcription factor for the development of regulatory CD4(+)CD25(+) T cells in the thymus, helped researchers to precisely phenotype most Tregs [42]. FoxP3 (also known as Scurfin, IPEX, and JM2) is a transcriptional repression factor of the winged helix family and is expressed in all CD4(+) Treg cells with regulatory activity. Currently, Tregs may be accurately identified as CD4(+)CD25(+)FoxP3(+) T cells or (as FoxP3 inversely correlate with cell surface CD127 expression) as CD4(+)CD25(+)CD127(lo)/(−) T cells [43]. Specific regulatory T cell populations may also express other surface markers such as CD39, LAG-3 and GITR [44–47].

##### Natural and induced Tregs

Tregs can be subdivided into thymus-developed, “natural” Tregs that mediate tolerance to self-antigens and “induced” Tregs derived from conventional CD4(+) T cells following non-self antigenic exposure [48]. Natural Treg production requires stable expression of FoxP3 and high-affinity binding of HLA/self-peptide complex on thymic antigen-presenting cells (APCs) to T cell receptor (TCR). Natural Tregs can be also sub-classified into CD45RA(+)“naïve” Tregs and CD45RO(+)“memory” Tregs [49]. ‘Induced’ or ‘adaptive’ Tregs (iTregs) are generated from naïve T cells in the presence of transforming growth factor-β (TGF-β) or retinoic acid and produce the anti-inflammatory cytokine IL-10 [50–52]. Despite the phenotypic and functional overlaps with natural Tregs, iTregs demonstrate apparent differences in stability and gene expression [53]. Type 1 regulatory T cells (Tr1), are a subpopulation of Tregs-expressing CD4(+)CD49(+)LAG-3(+)IL-10(+) that exert significant immunosuppressive effects [54–56]. In addition, CD8(+) Tregs (Tr2) and IL-17-producing Tregs that share some common features with Tr1 also exist [57]. iTregs-expressing RORγt, which is the master regulator of antimicrobial type 3 immunity are termed type 3 Tregs (Tr3) [58, 59]. These cells constitute the major population of colonic Tregs, require bacterial antigens for differentiation and are distinct from thymus-derived Tregs.

##### Tregs function

Tregs have a pivotal function in regulating the immune system by controlling the number and function of effector cells. Thus, they play a major role in suppressing unwanted autoreactive immune responses, such as in the case of autoimmunity [60]. Interestingly, it has been indicated that Tregs can modulate both adaptive and innate immune systems, and once activated they specifically regulate immune responses at multiple levels and by various mechanisms. These suppressive mechanisms can be organized into major groups, including cell–cell contact-dependent suppression, inhibitory cytokine release (such as IL-10 and TGF-β), modulation of APC function, cytolysis, metabolic disruption and induction of suppressor cells or “infectious tolerance” [53].

In addition to IL-10, the inhibitory cytokine IL-35 also contributes to regulatory T cell function [61, 62]. IL-35 belongs to IL-12 family of cytokines that includes IL-12, IL-23, IL-27 and IL-35. Of these, IL-12 and IL-23 have pro-inflammatory roles, whereas IL-35 appears to exert a more regulatory function by inducing the expansion of Tregs and Bregs subsets and inhibiting Th17 cell differentiation [63]. IL-35-producing Tregs represent a distinct effector population from the IL-10-producing iTregs which also have different transcription factor dependency, as differentiation regulator Blimp1 is essential for IL-10 production, but not for IL-35, whereas Foxp3 is important for IL-35 but dispensable for IL-10 production [64]. Recently, it was demonstrated that the IL-12p35 alpha subunit of IL-35 efficiently suppressed encephalitogenic T cell responses and ameliorated experimental autoimmune encephalomyelitis (EAE), a well-characterized murine model of MS [65]. IL-12p35 inhibited the expansion of pathogenic Th17 and Th1 cells and mediated the expansion of Tregs and Bregs [65].

#### Tregs and multiple sclerosis

Major studies investigating the role of Tregs in MS are summarized in Table 1.

**Table 1:** Main studies investigating the effect of MS-treated patients on regulatory B and T cells

Authors, year of study	Origin/country	Treatment	Sample	Results
Quan et al. (2015)	China	Rituximab	Healthy controls (*n* = 19) NMO patients (*n* = 9)	Tregs increased from 0.3 to 1.2% of total lymphocytes after 48 weeks
De Mercanti et al. (2016)	Europe	Alemtuzumab	RRMS patients (*n* = 29)	Significant increase in CD4(+)CD25(hi)CD127(lo)FoxP3(+) Tregs after 24 months of treatment
Haas et al. (2015)	Germany	Fingolimod	Healthy controls (*n* = 37) MS patients (*n* = 74)	Increased median percentage of Tregs from 3 to 6,7% after 3 months of treatment
Blumenfeld et al. (2016)	Israel	Fingolimod	MS patients (*n* = 10)	Increase in the percentage of CD38(hi)CD24(hi) “transitional” Bregs from 3.7 to 11.6%
Piancone et al. (2016)	Italy	Fingolimod	RRMS patients (*n* = 12)	Significant increase in CD19(+)BTLA(+)IL-10(+) B cells both as a percentage of total lymphocytes and CD19(+) B cells
Lundy et al. (2016)	USA	Dimethyl Fumarate	RRMS patients (*n* = 13)	After 12 months of treatment: CD19(+) B cells concentration was halved and CD24(hi)CD38(hi) Bregs were doubled
Stenner et al. (2008)	Germany	Natalizumab	RRMS patients (*n* = 15)	No significant change in Tregs percentage 30 days after initiation of therapy
Putzki et al. (2010)	Switzerland	Natalizumab	RRMS patients (*n* = 28)	Relative decrease in CD4(+)CD25(+) Tregs from 18.9 to 14.1%
Schubert et al. (2015)	USA	IFN-β	Treatment-naïve RRMS patients (*n* = 10) IFN-β-treated RRMS patients (*n* = 11)	Increase in CD24(hi)CD38(hi) “transitional” Bregs from 1.09 to 9.50%
Ireland et al. (2014)	USA	Glatiramer acetate	Treatment-naïve MS patients (*n* = 22) Glatiramer acetate-treated MS patients (*n* = 22)	Treated patients IL-10 production by B cells was equivalent to those in healthy donors and up to 6.5-fold greater than the levels in treatment-naive patients

##### EAE mouse model

The role of Tregs in MS has been thoroughly investigated in EAE [66]. EAE shares many features with the human disease and has thus revealed much information that led to the development of many approved therapies for MS [67, 68]. A correlation was found between antigen-specific Tregs and disease resistance [69]; similarly, transfer of Tregs to EAE-induced mice reduced the severity of the disease [70]. In addition, depletion of CD25(+) T cells reduced the antigen burden required to induce EAE and prevented disease recovery [71]. Furthermore, Tregs were also involved in the regulation of cell transmigration across the BBB [72]. The EAE model system was also exploited for in vivo silencing of certain microRNAs such as miR26a, which increases the expression of Th17-related cytokines and establishes more severe EAE [73]. In contrast, overexpression of miR26a is associated with decreased expression of Th17-related cytokines, positive correlation with Treg FoxP3 and less severe disease [73].

Many treatment regimens increase Tregs and have also been proven quite successful at the experimental level (in EAE) [74]. For instance, IDO was shown to upregulate Tregs via tryptophan catabolite and to suppresses encephalitogenic T cell responses [75]. Further evidence suggested that vitamin A and its active metabolites (all-trans-retinoic acid and 9-cis-retinoic acid) could restore the imbalance of Th17 and Treg cells and can be considered as a promising target in the prevention of EAE [76]. Expansion of Tregs also appears promising. Lately, it was shown that engineered clonal MBP-specific Tregs ameliorated EAE in myelin oligodendrocyte glycoprotein (MOG)-immunized DR15 transgenic mice [77]. Administration of antigen encapsulated within tolerogenic nanoparticles (tNPs) comprising biodegradable polymer is also capable of inducing Ag-specific Tregs [78]. tNP-treated mice did not develop EAE following adoptive transfer of encephalitogenic T cells [78].

##### Human MS

It has become apparent that Tregs are also implicated in the pathophysiology of MS in humans [53, 74]. Although increased frequencies of Tregs are found in the cerebrospinal fluid but not peripheral blood of MS patients [79], alterations in Treg homeostasis [80, 81] and their functional impairment are documented [82–84]. Interestingly, their functional defects are more profound in RRMS than in SPMS [85, 86]. Analysis of the thymic export activity in MS patients revealed impaired release of newly formed T cells into the periphery resulting in an imbalance of circulating Tregs [87]. This thymic functional impairment is compensated by peripheral post-thymic expansion, creating a shift from naïve Tregs to memory Tregs in MS patients. Researchers argue that this shift may account for the impaired suppressive function of Tregs in MS.

Another possible mechanism for the functional failure of Tregs appears to be pro-inflammatory cytokines, such as IL-12, which are up-regulated in MS [88]. IL-12 has the ability to change the phenotype and function of Tregs by inducing IFN-γ production. IFN-γ-producing Tregs display a decline in their suppressive activity in vitro, as IFN-γ blockade significantly boosted their suppressive ability but did not affect control Tregs [89]. The increased percentages of Th1-like Tregs may partly account for the lack of suppressive function Tregs of MS patients. These data illustrate the phenomenon of enhanced Tregs plasticity toward a pro-inflammatory, cytokine-producing effector phenotype [90]. Skewed IFN-γ-producing Th1-like Tregs play significant role in MS and also other autoimmune diseases [53, 91] and malignancies [92]. Importantly, IL-12 dependent IFN-γ production of Tregs could also be mimicked in vitro in Tregs from healthy subjects creating Th1-like Tregs that resembled a classical Th1 phenotype [89]. Moreover, Th17-like Tregs which expanded in the presence of IL-6 and IL-1β have also been documented [93, 94].

Apart from FoxP3(+) Tregs, the role of Tr1 cells’ role in MS seems to be equally important. In MS, Tr1 cells were reduced in CD46-activated T cells [83], which are known to acquire a Tr1 phenotype [95]. Furthermore, IL-10 production from CD46- activated T cells was almost absent, while IFN-γ production was not affected in these cells. It can, therefore, be concluded from these findings that MS is associated with multiple defects in regulatory T cell populations [83].

#### Bregs

Bregs are a subset of B cells that display suppressive functions toward pro-inflammatory and autoreactive immune responses. They express IL-10 and other regulatory cytokines such as TGF-β and IL-35 that limit inflammation [63, 96–98]. On the other hand, an overly suppressed immune system is vulnerable to infection or cancer, so a balanced number and function of Bregs is essential [99, 100].

#### Phenotype

Despite extensive research on Bregs in recent years, to date there is no consensus on a specific Breg phenotype [101]. Although there is a number of identifiable Breg subsets, it is still not known if they are developed from a distinct cell lineage, like thymus-derived Tregs, or are induced by immunological triggers [102]. On a similar note, no Breg-specific transcription factor has been identified. Although expression of Foxp3 by certain Bregs is documented [103], it is still a matter of debate whether B cell-specific expression of FoxP3 facilitates acquisition of suppressive capacity. Due to the lack of molecular markers, Bregs are usually identified by their ability to express IL-10, and these are termed B10 cells. In humans, both “naïve” CD19(+)CD24(hi)CD38(hi) [104] and “memory” CD19(+)CD24(hi)CD27(+) [105] Bregs have been identified as the principal IL-10-expressing subsets [106]. Bregs also express high levels of CD5 [107–109], while CD5(+)IL-35-producing Bregs and TGFβ-producing Bregs have also been described [110, 111]. Furthermore, B cells with regulatory function can also express CD25 and LAG-3, similarly to Tregs [112, 113]. Moreover, Bregs such as CD73(−)CD25(+)CD71(+) BR1 subsets of plasma cells are associated with anti-inflammatory IgG4 antibody secretion which is important for allergic airway inflammation [114, 115]. This illuminates a further immune-regulatory role of the non-inflammatory and blocking antibody function of IgG4, which may require further investigation.

#### Bregs function

Bregs suppress the pro-inflammatory cytokine production by dendritic cells, leading to inhibition of Th1 and Th17 differentiation [116]. Bregs have also been documented to support in vivo Tregs expansion in both mice and humans [104, 117]. In several reports, flow cytometry and/or magnetically sorted CD4(+) T cells were co-cultured with autologous Bregs to elucidate the effector mechanisms of Bregs on CD4(+) T cells, including the generation of suppressive Tregs [118–120]. In co-culture with Bregs, CD4(+)CD25(−) T cells produced less IFN-γ and IL-17, whereas Treg induction was predominantly facilitated by expression of IL-10 and TGF-β. All these studies confirmed previous data from murine systems where naïve T cells co-cultured with a mixture of APCs consisting of B and dendritic cells in the presence of TGF-β, retinoic acid and IL-2, differentiated into allogeneic Tregs [121]. In addition, through the expression of TGF-β, lipopolysaccharide (LPS)-activated B cells can promote both the apoptosis of CD4(+) [122] and anergy in CD8(+) [123] effector T cells.

- *B10* B10 is a Breg subset whose regulatory function is entirely attributed to their IL-10 production. Moreover, this suppressive function seems to be antigen specific, most likely due to antigen-specific B cell receptor (BCR) signaling [124, 125]. This BCR specificity explains the rapid B10 response to antigens, self- or otherwise, rendering them capable of suppressing unwanted excessive immune responses [reviewed by [126] ].
- *IL*-*10 independent Bregs* A novel CD138(+)IL−35(+) Breg (i35 Breg) population has been characterized recently, which produces IL-35, apart from IL-10. Through IL-35 expression, these cells regulate CNS inflammation. IL-35 has the ability to transform conventional B cells or B10 cells to IL-35-expressing i35-Bregs [reviewed by [127] ]. Furthermore, TGF-β-expressing Bregs are thought to play a role in the suppression of allergic reactions. They evidently promote Treg differentiation by upregulating FoxP3 production in T cells and regulate food allergy-induced inflammation in mice. In addition, thrombospondin 1-secreting CD35(+) B cells induced a Treg phenotype through TGF-β, but not IL-10 and suppressed co-stimulatory molecule expression on dendritic cells. Moreover, there is evidence that PD-L1 (programmed death 1) is involved in Bregs function, as PD-L1^Hi^ B cells negatively regulate T cell differentiation [128] (reviewed by [129]).
- *BTLA*-*expressing Bregs* B and T lymphocyte attenuator (BTLA or CD272) is an immunoglobulin, which, like programmed death-1 (PD-1), is involved in the suppression of immune responses. BTLA contains two immunoreceptor tyrosine-based inhibitory motifs (ITIM) and is expressed on a wide range of immune cells including T and B lymphocytes, NKT cells, NK cells, macrophages, dendritic cells [130] and follicular Th1 cells [131].

#### Bregs and multiple sclerosis

##### EAE mouse model

B cells can play a regulatory role in EAE pathophysiology, as mice with genetically deficient B cells cannot recover from the disease, whereas transfer of IL-10-producing B cells suppresses EAE symptoms [124, 125]. For instance, Bregs, transduced into mice with EAE, accumulated in the spleen and mesenteric lymph nodes, leading to an expansion of Tregs and Tr1 cells in vivo [132]. Importantly, Tregs and Tr1 s were also enriched in the CNS of the same littermates. In the EAE model again, treatment with MOG protein fused to reovirus protein σ1 (MOG–pσ1), resulted in an expansion of IL-10-producing B220(+)CD5(+) Bregs, which restored Tregs and facilitated the rapid improvement of EAE [133]. Additionally, PD-L1^Hi^ Bregs transferred to afflicted animals suppressed the disease. In total, Bregs, in contrast to effector B cells, protect from the development of EAE, by suppressing pro-inflammatory cytokines and the transmigration of activated cells to the CNS [97, 134, 135].

##### Human MS

**Fig. 1:**
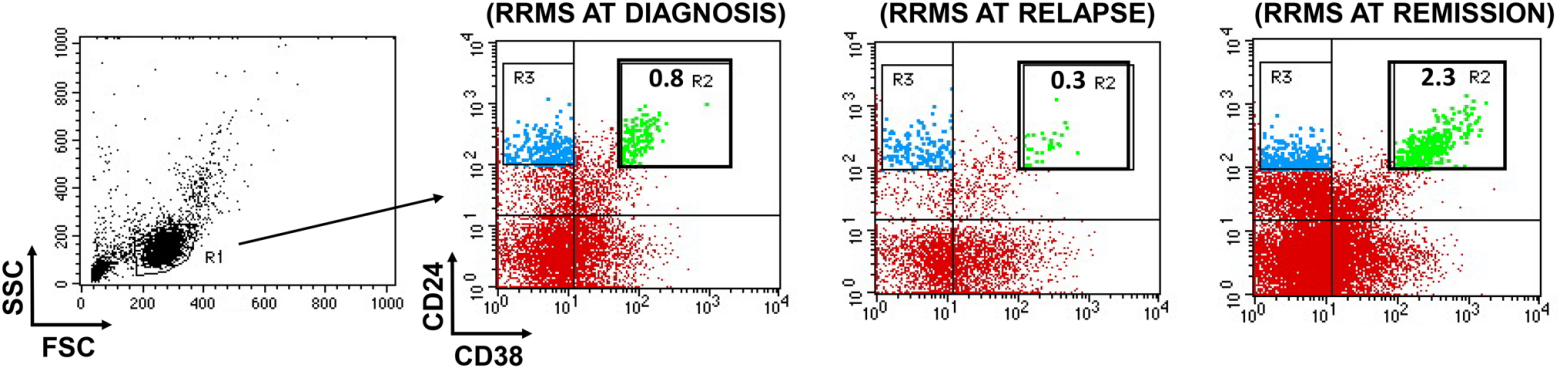
Typical flow cytometric analysis of memory and transitional Bregs in RRMS. PBMCs from representative patients with RRMS at diagnosis, relapse and remission were stained with CD19, CD24, CD27 and CD38 moAbs and analyzed by flow cytometry. Total lymphocytes were gated based on forward-side scatter characteristic excluding dead cells and debris (gate R1). Transitional Bregs were identified based on high expression of CD38 and CD24 markers (green color—gate R2) and positivity for CD19. Memory Bregs were identified based on high expression of CD24, positivity for CD19, CD27 markers and lack of CD38 expression (blue color—gate R3). At remission, transitional Bregs appear significantly increased

There is no consensus on Breg numbers in autoimmune diseases. In most diseases or disease states, Bregs are reduced [136–140] but increased numbers were also reported [105]. In MS in particular, Bregs are reported to be numerically decreased [141, 142], unaltered [143, 144] or increased [145]. A representative phenotypic flow cytometric analysis of Bregs in RRMS is shown in Fig. 1. Irrespective of their numbers, Bregs function is impaired in MS patients, as IL-10 production and suppressive function of B cells are reduced [21, 146–148]. In addition, the proportion of naïve Bregs in disease relapses is reduced, leading to an increased memory/naïve ratio [141]. Whether this reduction is the cause or the consequence of disease relapse remains to be seen. Recent data also have indicated that reduced peripheral blood Breg levels were not associated with the Expanded Disability Status Scale score in MS [149].

A novel type of Bregs, CD19(+)CD25(+) cells, was described in both healthy subjects and MS patients [112, 145]. This new subtype seems to be numerically increased in MS compared to healthy controls, and also in relapse compared to disease remission [135]. It is apparent that much more research is needed to illuminate the role of different Breg subsets in MS [150].

### Effects of MS therapies on Tregs and Bregs

#### Monoclonal antibodies

##### Anti-CD20

B cell-depleting therapies in MS focus on two main targets, CD20 and CD19. Monoclonal anti-CD20 includes rituximab, ocrelizumab and ofatumumab, which differ in their CD20 epitope recognition and in the intensity of their action [151]. CD20 is expressed on most B cells, from PreB to IgG memory B cells, while leaving plasmablasts and ProB cells mostly lack expression. All anti-CD20 therapies cause an almost complete extinction of B cell subtypes in peripheral blood [152]. B cell repopulation begins several months post-treatment and appears to be inclined toward a more naïve and regulatory phenotype [153]. B cell depletion also suppresses T_H_1 and T_H_17 responses and increases circulating Tregs. In general, this therapy has shown to ameliorate disease symptoms and activity, and reduce relapse rate, including reduction in gadolinium-enhancing lesions on brain MRI [154, 155].

##### Anti-CD19

Anti-CD19 monoclonal therapy also looks promising. IgG1 anti-CD19 antibody (MEDI-551) [156] recently entered a phase II clinical trial in MS. CD19 is expressed on all B cells and is progressively lost in terminally differentiated plasma cells [157]. As a result, it comes as no surprise that MEDI-551 induced a longer-lasting B cell depletion than rituximab, while also reducing immunoglobulin serum levels, including autoantibodies [155]. Anti-CD19 therapy in EAE-induced mice suppressed disease severity and duration and increased circulating Tregs, whereas potentially protective CD1d(hi)CD5(+) Bregs displayed resistance to depletion [158]. Hence, MEDI-551 is expected to have similar effects to anti-CD20 therapy on MS patients and could be approved for the treatment of MS in the future especially targeting autoreactive CD19(+)CD20(−) plasma cells that would be resistant to CD20 mAb treatment [159].

##### Alemtuzumab

Alemtuzumab is a humanized IgG1 monoclonal antibody that targets the CD52 (Campath-1 antigen), a 12 amino acid glycoprotein anchored to glycosylphosphatidylinositol, which is widely expressed on the cell surface of mature immune cells. Anti-CD52 induces a rapid and prolonged depletion of lymphocytes from the circulation, which results in a profound immunosuppression status followed by an immune reconstitution phase [160]. In EAE, anti-CD52 treatment abrogated B cell infiltration and disrupted existing B cell aggregates in the CNS [161]. Recently, it has been shown that it can also ameliorate colitis through suppressing Th1/17 mediated inflammation and promoting Tregs differentiation in IL-10 deficient mice [162]. Much of the research on alemtuzumab focuses on the lymphocyte repopulation progress and shows that CD4(+)CD25(+)CD127(low) Treg cells preferentially expand within the CD4(+) lymphocytes, reaching their peak within 1 month [163]. A recent study demonstrated that alemtuzumab increased anti-inflammatory cytokines (such as IL-10 and TGF-β) while diminishing pro-inflammatory cytokines (such as IFN-γ, IL-17, IL-6 and TNF-α) within 6 months of treatment and increased Tregs percentage and function after 24 months post-treatment [164]. In addition, alemtuzumab seems to affect B cells, as it increased the percentage of repopulated naïve/immature B cells [165, 166]. Alemtuzumab may thus be a promising therapy for MS [6]; however, it also causes loss of immune-tolerance leading to secondary autoimmunity such as Graves’ disease and marked anti-drug antibody responses [160, 167].

##### Natalizumab

Natalizumab, approved for the treatment of MS, is a humanized IgG4 monoclonal antibody. It mainly binds to the α4-chain of α4β1 integrin heterodimer—also known as very late activating antigen-4 (VLA-4), on the surface of leucocytes and inhibits binding of VLA-4 to vascular cell adhesion molecule-1 (VCAM-1) and, consequently, the attachment of leucocytes to the inner lining of cerebral vascular walls and their crossing of the BBB. This crossing directly diminishes IgM and partially IgG in the CSF [168] as well as in the serum [169]. Thus, natalizumab modulates B cell functions, but appears to be unable to restore the suppressive function of Tregs while marginally decreasing their percentages in MS [170, 171].

##### Tocilizumab

Tocilizumab is a humanized IgG1 anti-IL-6 receptor monoclonal antibody approved by the FDA for the treatment of rheumatoid arthritis, active systemic juvenile idiopathic arthritis and polyarticular juvenile idiopathic arthritis. IL-6 is known to induce plasmablast production of anti-aquaporin 4 (Aqp-4) antibodies in vitro and may account for neuromyelitis optica (NMO) disease activity [172]. IL-6 concentration is increased in the CSF of NMO patients [173] and IL-6 induces pro-inflammatory Th17 cells in both NMO and MS patients [174]. Thus, tocilizumab modulates Th17 cells and plasmablasts. Although there are no data on its effect on Tregs and Bregs, tocilizumab appears to be an attractive candidate therapeutic agent for MS.

#### Other immunomodulatory agents

##### Fingolimod

Fingolimod is an approved therapeutic agent for RRMS. It has a potent pharmacological action because it functions as an unselective agonist of sphingosine 1-phosphate receptors (S1PR) and as a selective antagonist of the S1P1 subtype by induction of receptor downregulation [175]. Since S1P1 is fundamental in the regulation of lymphocyte trafficking, its downregulation leads to redistribution of the immune cells to secondary lymphoid tissues, resulting in the depletion from the circulation and therefore immunosuppression [175]. It prevents lymphocyte egress from secondary lymphoid tissues, promoting loss of CCR7-expressing T cells and increase in Treg numbers and their suppressive function on T cell proliferation [176, 177]. It may affect B cells, as it leads to increased percentage of plasma cells and a shift toward a more naïve and transitional B cell phenotype. In addition, treatment with fingolimod increases both Breg numbers and function, indicated by a boost in IL-10 production [8]. According to recent data, fingolimod significantly enhances CD19(+)BTLA(+)IL−10(+) B cells in RRMS patients, which may relate to amelioration of symptoms [142].

##### Dimethyl fumarate

Dimethyl fumarate (DMF) is an approved therapeutic agent for MS. Its in vivo metabolite monomethyl fumarate (MMF) can bind to brain endothelium cells leading to activation of nuclear factor (erythroid-derived 2)-related factor 2 (Nrf2) and downregulation of vascular cell adhesion molecule 1 (VCAM-1) [178].This can be mediated via the G-protein-coupled receptor (GPCR) hydroxycarboxylic acid receptor 2 (HCA2), a known molecular target of MMF. Studies have documented the binding of DMF to HCA2 on dendritic cells followed by the inhibition of pro-inflammatory cytokines production in vitro and in MS murine models [179]. Although its precise mechanism of action remains unclear, evidence indicates that activation of HCA2/GPR109A pathway can decrease immune responses and may enhance anti-inflammatory functions in the intestinal mucosa, possibly leading to reduction in CNS tissue damage in MS patients [180]. In addition, it causes depletion of circulating lymphocytes in peripheral blood [181, 182]. More precisely, DMF alters lymphocyte subsets homeostasis in MS patients, decreasing absolute lymphocyte counts, but does not affect all subsets uniformly [183]. CD8(+) T cells are mainly affected, with reductions in the CD4(+) cells, particularly within the pro-inflammatory T-helper Th1 and Th17 subsets also occurring, creating a bias toward more anti-inflammatory Th2 and regulatory subsets [183]. Both naïve and memory B cells were diminished in certain patients, while Bregs were increased after 4–6 months of therapy and remained in higher numbers at 12 months post-treatment. Also, IL-10 production was elevated in some patients [184]. Other studies showed a skewing from memory CD8(+) and CD4(+) T cells toward their naïve counterparts together with a curtail on T_H_1 cells in dimethyl fumarate-treated RRMS patients [9] and an anti-Inflammatory shift in B Cell subsets [183, 185]. These limited data appear very promising.

##### Teriflunomide

Teriflunomide is an approved oral therapeutic agent for MS relapses. Its main mechanism of action involves the suppression of the de novo synthesis of pyrimidines in rapidly proliferating cells such as T and B lymphocytes [186]. Pyrimidine synthesis inhibition leads to halt of the cell cycle in G1 phase and it thus has anti-proliferative results, reducing autoantigen-specific immune responses. In a recent study of teriflunomide in murine EAE, a significant increase in CD39(+) Treg concentration was observed, along with decrease in APCs of Peyer patches [187] [reviewed by [10] ].

##### Glatiramer acetate

Glatiramer acetate (GA), a random polymer consisting of four amino acids of the myelin basic protein, is considered a first-line treatment for MS. It prevents disease relapses and patient disability. This agent shifts T cells from a T_H_1 to a T_H_2 response [188, 189]. GA induces a Treg phenotype and increases FoxP3 expression while restoring Treg function [190]. B cells from GA-treated EAE mice also increased production of IL-10 and reduced expression of co-stimulatory molecules [191]. Importantly, the therapeutic effect of GA in EAE was abrogated in B cell-deficient mice [191, 192]. Another study demonstrated that B cell IL-10 expression was restored, and IL-6 production was diminished after glatiramer acetate treatment. There was also altered proliferation in response to CD40L and an increased immunoglobulin production by B cells [193].

##### Ifn

IFNβ-1b and IFNβ-1a are disease-modifying agents for RRMS, affecting multiple immunological processes. IFNβ suppresses the ability of APCs to present antigens and stimulate T cells [194] and prevents T cells from crossing the BBB, while channeling autoreactive T cells into lymphoid tissues [195]. In addition, IFNβ has the ability to induce Tregs, probably due to a shift to Treg-promoting cytokines, such as IL-4, IL-5 and IL-13 [195]. Transitional Bregs are thought to increase as well, as a result of IFN-β therapy [24, 196]. Moreover, the treatment causes Th17 death [197], reduces TNF and increases IL-27 production, known to slow down EAE progression. Thus, IFN-β therapy both impedes pro-inflammatory cells and cytokines and promotes anti-inflammatory ones in MS (Reviewed by [198]).

##### Statins

Apart from specialized therapies, there are other agents with immunomodulatory properties that could prove useful as supportive and/or complementary treatments for MS. One example is HMG-CoA reductase inhibitors (statins), which are a class of lipid-lowering medications known to have immunomodulatory properties. For instance, atorvastatin increases Tregs and reduces clinical disease activity in patients with rheumatoid arthritis. It also displayed anti-inflammatory effects on peripheral blood [199]. In a recent study, atorvastatin and lovastatin enhanced Tregs numbers, but also FoxP3 mRNA levels 30 days post-treatment. However, Treg numbers returned to standard levels after 45 days of treatment. Nevertheless, increased values of TGF-β, FoxP3, CTLA-4 and GITR-expressing Tregs were observed [200]. Simvastatin also regulated TGF-β signal transduction, leading to an increase of Tregs [201], and reduced pro-inflammatory cytokines in patients with rheumatoid arthritis. Statins have an effect on MS as well. Simvastatin suppressed mononuclear cell responses, reduced IFN-γ, TNF-α and IL-2 production and inhibited the antigen-presenting capacity of macrophages [202]. Furthermore, atorvastatin combined with glatiramer acetate showed synergistic immunomodulatory effects in MS [203].

##### Vitamin D

Vitamin D or 25-hydroxy vitamin D (25(OH)D)—the main vitamin D metabolite measured in blood—is known to have immunomodulatory properties. Vitamin D affects both B and T lymphocytes. It inhibits T cell proliferation and reduces IFN-γ, IL-2 and IL-17 expression [204], while increasing IL-10 and Tregs [205]. It also inhibits plasma cell production and increases IL-10-Bregs. Recent evidence from the EAE mouse model indicated that vitamin D-induced dendritic cells could ameliorate symptoms by enhancing the proportions of regulatory lymphocytes and reducing T-helper type 1 and type 17 cells [206]. Vitamin D deficiency is associated with an increased incidence of MS [207]. During MS relapse, 25(OH)D levels are generally decreased [208]. In a small study, vitamin D supplementation led to a significant reduction of the number of newly active brain lesions [209] (reviewed by [210]).

## Conclusion

Multiple sclerosis is the most prevalent autoimmune disease of the CNS and a frequent cause of neurological disability in young adults. As there is no cure for the disorder, the aim of new treatments is the alleviation of symptoms and the reduction of relapses. As with most autoimmune diseases, MS patients exhibit impaired immunoregulatory mechanisms that lead to harmful immune responses. It is not yet recognized whether this dysregulation is the cause or a consequence of the disease. Nevertheless, regulatory mechanisms play a major role in MS. Thus, it comes as no surprise that most if not all of MS therapies have immunomodulatory actions. It is important to conduct more research on current medications and their influence on regulatory lymphocytes to uncover their exact mechanism of action and to be able to administer the appropriate therapeutic agent to each patient, according to their particular condition (personalized or precision medicine). On a final note, other agents that are not currently in use in MS but have immunomodulatory properties, such as vitamin D or statins, could be beneficial as a complementary treatment for MS.

## Abbreviations

Aqp: Aquaporin
APC: Antigen-presenting cell
BBB: Blood–brain barrier
CSF: Cerebrospinal fluid
EDSS: Expanded disability status scale
ELISpot: Enzyme-linked immunospot
FACS: Fluorescence-activated cell sorting
FoxP3: Forkhead box P3
IFN: Interferon
mAb: Monoclonal antibodies
MBP: Myelin basic protein
MS: Multiple sclerosis
NMO: Neuromyelitis optica
PB: Peripheral blood
PBMC: Peripheral blood mononuclear cells
PPD: Purified protein derivative of tuberculin
RORC: Retinoid-related orphan receptor g
RRMS: Relapsing–remitting MS
SPMS: Secondary progressive MS
Tbet: T-box expressed in T cells
TGF: Transforming growth factor
Th: T-helper
TNF: Tumor necrosis factor
Treg: T-regulatory cells
VLA: Very late antigen
